# Knockout of KLF10 Ameliorated Diabetic Renal Fibrosis via Downregulation of DKK-1

**DOI:** 10.3390/molecules27092644

**Published:** 2022-04-20

**Authors:** Yung-Chien Hsu, Cheng Ho, Ya-Hsueh Shih, Wen-Chiu Ni, Yi-Chen Li, Hsiu-Ching Chang, Chun-Liang Lin

**Affiliations:** 1 Department of Nephrology, Chang Gung Memorial Hospital, Chiayi 613, Taiwan; t1608@cgmh.org.tw (Y.-H.S.); chu_chu73@yahoo.com.tw (W.-C.N.); kame769@cgmh.org.tw (Y.-C.L.); bet0529@cgmh.org.tw (H.-C.C.); 2 Kidney and Diabetic Complications Research Team (KDCRT), Chang Gung Memorial Hospital, Chiayi 613, Taiwan; hc1238@cgmh.org.tw; 3 Division of Endocrinology and Metabolism, Chang Gung Memorial Hospital, Chiayi 613, Taiwan; 4 School of Traditional Chinese Medicine, College of Medicine, Chang Gung University, Taoyuan 333, Taiwan; 5 Kidney Research Center, Chang Gung Memorial Hospital, Taipei 105, Taiwan; 6 Center for Shockwave Medicine and Tissue Engineering, Chang Gung Memorial Hospital, Kaohsiung 833, Taiwan

**Keywords:** diabetes, renal fibrosis, KLF10, DKK-1

## Abstract

Diabetes-induced chronic kidney disease leads to mortality and morbidity and thus poses a great health burden worldwide. Krüppel-like factor 10 (KLF10), a zinc finger-containing transcription factor, regulates numerous cellular functions, such as proliferation, differentiation, and apoptosis. In this study, we explored the effects of KLF10 on diabetes-induced renal disease by using a KLF10 knockout mice model. Knockout of KLF10 obviously diminished diabetes-induced tumor growth factor-β (TGF-β), fibronectin, and type IV collagen expression, as evidenced by immunohistochemical staining. KLF10 knockout also repressed the expression of Dickkopf-1 (DKK-1) and phosphorylated β-catenin in diabetic mice, as evidenced by immunohistochemical staining and Western blot analysis. Quantitative reverse transcriptase–polymerase chain reaction (RT-PCR) revealed that significantly decreased type IV collagen, fibronectin, and DKK-1 existed in KLF10 knockout diabetic mice compared with control diabetic mice. Moreover, knockout of KLF10 reduced the renal fibrosis, as shown by Masson’s Trichrome analysis. Overall, the results indicate that depletion of KLF10 ameliorated diabetic renal fibrosis via the downregulation of DKK-1 expression and inhibited TGF-β1 and phosphorylated β-catenin expression. Our findings suggest that KLF10 may be a promising therapeutic choice for the treatment of diabetes-induced renal fibrosis.

## 1. Introduction

Diabetes mellitus is a global disease [1]. Several complications, such as cardiovascular disease [2], nerve damage (neuropathy) [3], and kidney damage (nephropathy) [1], occur in diabetic patients and increase the mortality of such patients. Diabetic nephropathy (DN), also called chronic kidney disease, leads to the development of end-stage renal disease (ESRD) [4]. ESRD, which eventually causes renal fibrosis, is characterized as the massive accumulation of extracellular matrix (ECM), impairment of the glomerular filtration rate, and increased albuminuria [5]. Current treatment of ESRD depends on dialysis or transplantation. However, accumulating research efforts have begun to focus on the development a new target to delay the progression of ESRD.

Upon binding to a specific receptor, transforming growth factor-β1 (TGF-β1) triggers the phosphorylation of Smads and regulates several cellular functions, such as cell differentiation, apoptosis, and immune homeostasis [6]. In chronic kidney disease, TGF-β1promotes renal fibrosis through canonical (Smads-dependent) and non-canonical (Smads-independent) pathways [7]. Activation of TGF-β1 enhances Smad2/3/4 and subsequently elevates fibronectin, collagen, and α-smooth muscle actin (α-SMA) [7]. In addition, TGF-β1 regulates mitogen-activated protein kinase (MAPK) and the phosphatidylinositol 3-kinase/protein kinase B pathway to elevate matrix formation and cell proliferation and migration, which are involved in renal fibrosis [7].

Krüppel-like factor 10 (KLF10), also referred to as the TGF-β inducible early gene (TIEG1), plays a critical role in TGF-β-induced signals [8]. By binding to a CG-rich sequence and through the zinc finger domain, KLF10 controls the genes involved in TGF-β-induced signals via the repression of Smad7 [9] and the activation of Smad2 [10]. Dysregulation of KLF10 has been observed in several human diseases, such as cardiovascular disease, skeletal muscle disease, and cancers [11]. Overexpression of KLF10 has been found in renal clear cell carcinoma [12]. Enhanced KLF10 expression coinciding with increased TGF-β1 has also been observed in high-fat and high-sucrose diet-induced non-alcoholic steatohepatitis [13]. Depletion of KLF10 increases the expression of collagen type I and fibronectin, leading to enhanced fibrosis in dystrophic skeletal muscle cells [14]. 

Another risk factor for the development of diabetes-induced renal fibrosis (DN) is the Wnt pathway [15]. The Wnt family, containing at least 19 secreted glycoproteins, can bind to its receptor frizzled (Fzd) protein and co-receptor low-density lipoprotein receptor-related protein-5 (LRP5) [16]. Activation of the Wnt pathway leads to nuclear accumulation of β-catenin and enhanced expression of fibronectin, the extracellular matrix component, and Snail to promote epithelial to mesenchymal transition, and, consequently, fibrosis in renal tissue [16]. Dickkopf (DKK) proteins function as a Wnt inhibitor via induced internalization of LRP5 and block the Wnt signal [17]. A previous report demonstrated that overexpression of DKK-1 attenuates β-catenin-induced renal fibrosis-related gene expression [18]. Dai et al. [19] demonstrated that elevated β-catenin is found in DN and focal segmental glomerulosclerosis as a result of podocyte dysfunction. In addition, DKK-1 alleviates Adriamycin-induced podocyte lesions through the downregulation of β-catenin activity [19]. Hou et al. showed that a low DKK-1 level is associated with high urinary albumin/creatinine ratios (UACRs) and albuminuria in diabetic patients [20]. 

A previous report from our laboratory indicated that a high glucose level elevates DKK-1 expression and phosphorylated β-catenin and mitigates the accumulation of β-catenin in the nucleus [21]. Knockout of DKK-1 reduces fibronectin expression in the entire kidney or glomerular mesangium in streptozotocin (STZ)-induced diabetic rats [21]. Killick et al. demonstrated that DKK-1 treatment significantly triggers KLF10 expression in primary neural cultured cells and causes Alzheimer disease [22]. However, no link between DKK-1 and KLF10 in DN has been reported. This study aims to investigate the effects of KLF10 on renal fibrosis-related proteins including TGF-β1 and DKK1 pathway expression in STZ-induced diabetes by using KLF10 knockout mice.

## 2. Results

### 2.1. Biochemical Properties of Experimental Animals

As shown in Table 1, STZ significantly increased the fasting blood glucose and hemoglobin A1c (HbA1C) level in mice. By contrast, their body weight was obviously reduced in response to STZ treatment. Interestingly, knockout of KLF10 improved renal functions, including reduced kidney weight and total protein/creatinine ratio in the urine of the STZ-treated group. These results indicate that STZ-induced diabetes and knockout of KLF10 reversed renal dysfunction.

### 2.2. Knockout of KLF10 and Its Downstream Targets in Diabetic Mice

**Figure 1:**
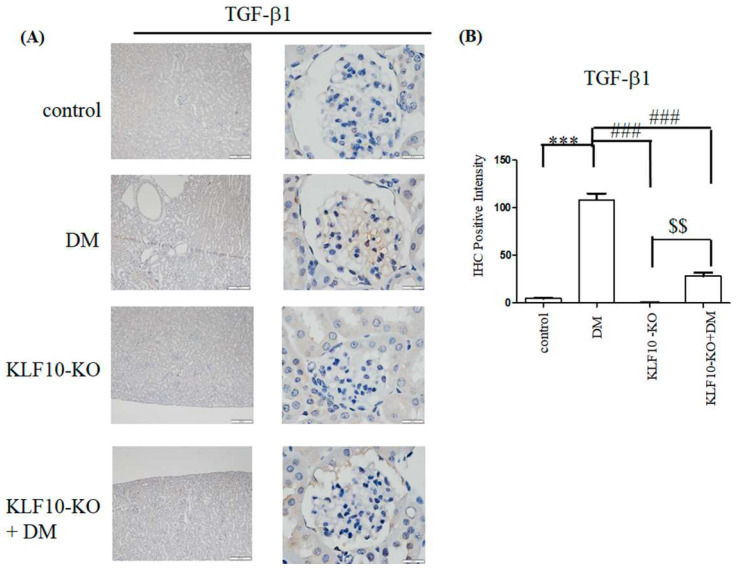
Decreased expression of TGF-β1 in KLF10 knockout (KLF10-KO) in diabetic (DM) mice. (**A**) Immunostaining or (**B**) RT-PCR analysis of kidney glomerular mesangium sections derived from control or KLF10-KO mice with or without diabetes (DM). The sections were subjected to immunostaining with TGF-β1 primary antibody. Left panel: magnification of 100×, right panel: magnification of 1000×. (**B**) Statistical analysis. Data are presented as means ± standard deviation from three different mice. ***: *p* < 0.001 compared with the control group. ###: *p* < 0.001 compared with the DM group. $$: *p* < 0.01 compared to KLF10-KO group. Scale bar: 200 μM at 100× and 20 μM at 1000×.

To investigate whether knockout of KLF10 influenced the TGF-β1 expression, immunohistochemical staining and quantitative RT-PCR were conducted. As shown in Figure 1, increased positive immunostaining signals (dark brown color) for TGF-β1were found in the diabetes group. Depletion of KLF10 repressed the expression of TGF-β1. Quantitative RT-PCR indicated that significantly increased TGF-β1 was observed in the diabetes group (3.28-fold) compared with the control group. The expression of TGF-β1 was obviously reduced to nearly that of the control group in the KLF10 knockout diabetic mice.

**Figure 2:**
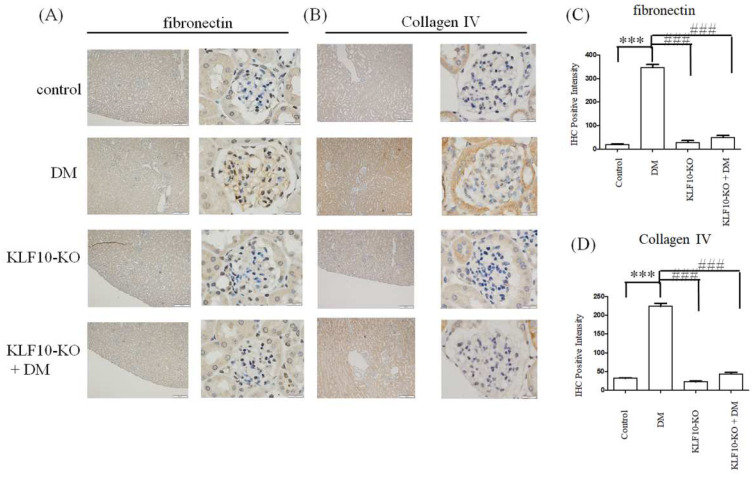
Knockout of KLF10 repressed fibronectin and type IV collagen expression in diabetic (DM) mice. Kidney glomerular mesangium sections derived from control or KLF10-KO mice with or without diabetes (DM) were subjected to immunostaining with fibronectin (**A**) and type IV collagen (**B**) primary antibody. Left panel: magnification of 100×, right panel: magnification of 1000×. (**C**) Fibronectin and (**D**) collagen IV; data are presented as means ± standard deviation from three different mice ***: *p* < 0.001 compared with the control group. ###: *p* < 0.001 compared with the DM group. Scale bar: 200 μM at 100× and 20 μM at 1000×.

TGF-β1 mediates fibronectin and type IV collagen expression and plays a critical role in diabetes-induced renal dysfunction [6]. To determine whether KLF10 knockout ameliorates TGF-β1-induced diabetic renal fibrosis, antibodies were used against renal fibrosis-related proteins. The expression of type IV collagen and fibronectin significantly increased in the diabetes group, whereas knockout of KLF10 repressed diabetes-induced type IV collagen and fibronectin expression (Figure 2).

**Figure 3:**
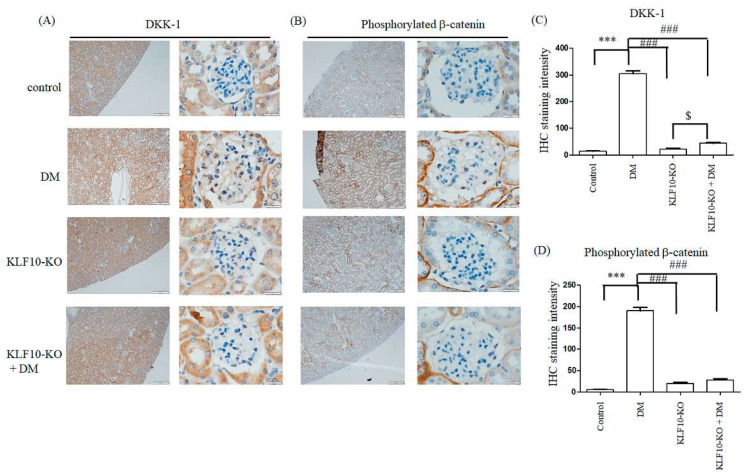
Knockout of KLF10 repressed DM-induced DKK-1 and phosphorylated β-catenin expression. Kidney glomerular mesangium sections derived from control or KLF10 knockout mice with or without diabetes (DM) were subjected to immunostaining with the (**A**) DKK-1 and (**B**) phosphorylated β-catenin primary antibody. Left panel: magnification of 100×, right panel: magnification of 1000×. (**C**) DKK-1 and (**D**) phosphorylated β-catenin; data are presented as means ± standard deviation from three different mice ***: *p* < 0.001 compared with the control group. ###: *p* < 0.001 compared with the DM group. $: *p* < 0.05 compared to KLF10-KO group. Scale bar: 200 μM at 100× and 20 μM at 1000×.

Previous reports have demonstrated that the loss of DKK-1, a β-catenin inhibitor, leads to the repression of TGF-β1 and fibronectin in diabetes-induced renal fibrosis [21]. Moreover, DKK-1 has been reported to regulate KLF10 expression [22]. To test whether KLF10 depletion also affects the expression of DKK-1 and phosphorylated β-catenin, we performed immunostaining analysis. Elevated DKK-1 and phosphorylated β-catenin were observed in the diabetes group compared with the control alone. Downregulation of KLF10 diminished the expression of DKK-1 and phosphorylated β-catenin in the diabetes group (Figure 3).

### 2.3. Effects of KLF10 Knockout on the Expression of Fibrosis-Related Genes

**Figure 4:**
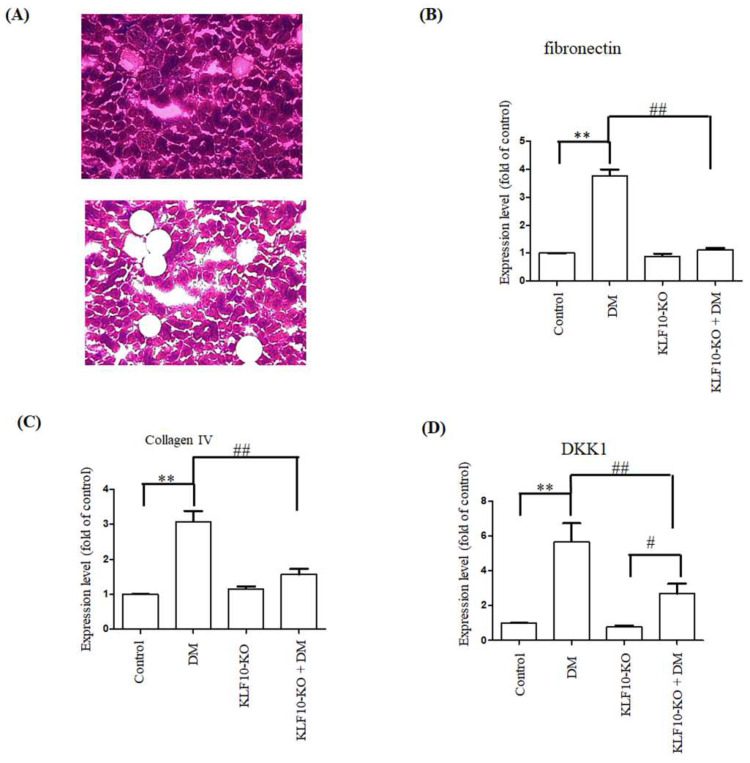
Repression of fibrosis-related gene expression in KLF10 knockout diabetic mice. (**A**) Images at ×100 magnification obtained before (upper panel) and after (lower panel) LCM capture. Total RNA extracted from glomerular mesangium derived from control or KLF10 knockout mice with or without diabetes (DM) were subjected to quantitative RT-PCR analysis of (**B**) fibronectin, (**C**) type IV collagen, and (**D**) DKK-1. Data are presented as means ± standard deviation obtained from at least three independent experiments. **: *p* < 0.01 compared with the control group. ##: *p* < 0.01 compared with the DM group. #: *p* < 0.05 compared to KLF10-KO group.

Quantitative RT-PCR was conducted to examine the regulatory effects of KLF10 on fibrosis-related gene expression. The gene expression of fibronectin, type IV collagen, and DKK-1 increased significantly to 3.76, 3.08, and 5.66 folds, respectively, in the DM group compared with the control group. No significant alternation of fibronectin and type IV collagen was found in the KLF10 knockout diabetes group compared with the KLF10 knockout alone. DKK-1 expression was significantly elevated in KLF10 knockout diabetes group compared with KLF10 knockout alone (*p* < 0.05). Moreover, the expression of fibronectin, type IV collagen, and DKK1 was significantly downregulated in KLF10 knockout DM compared with DM alone (Figure 4).

**Figure 5:**
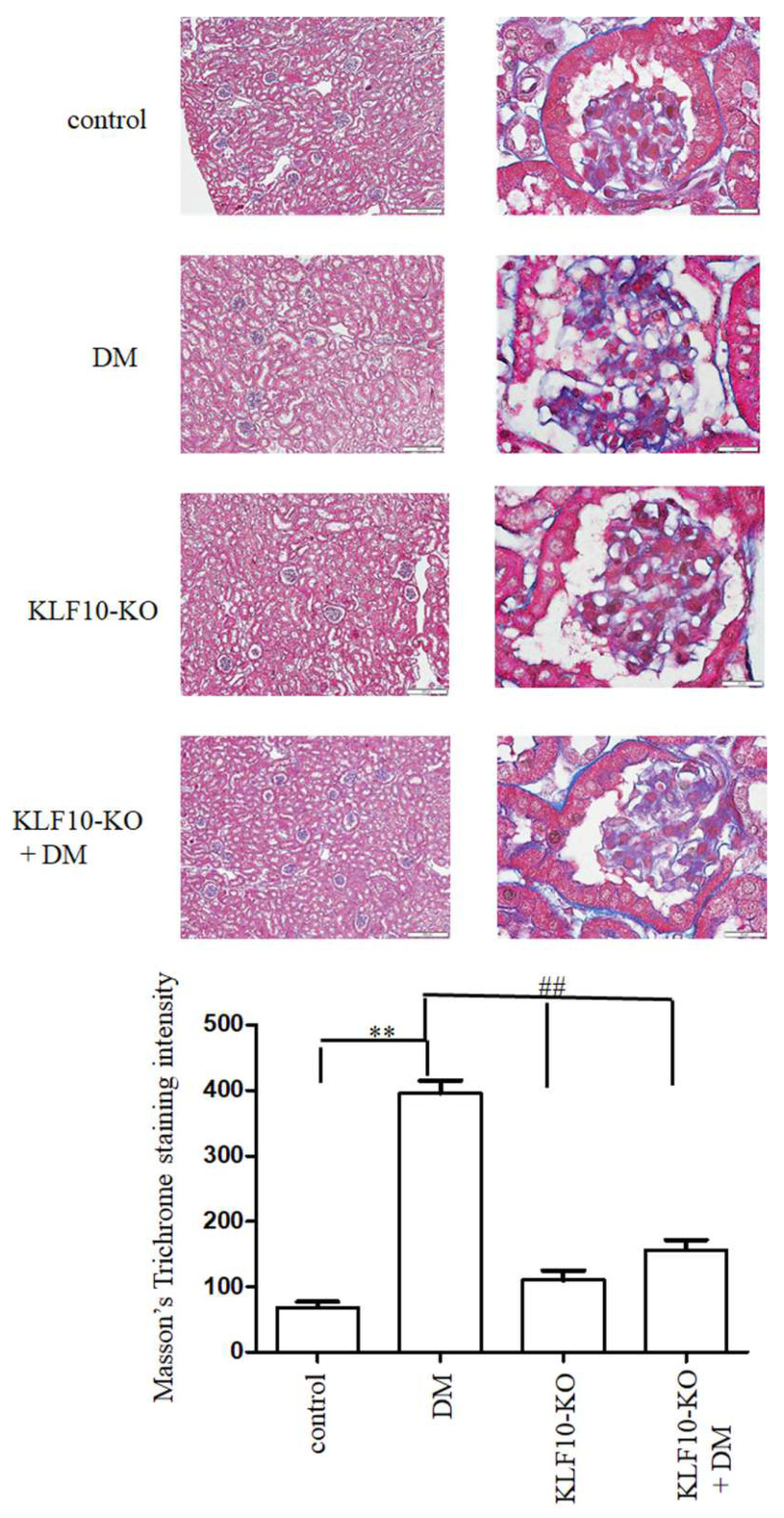
Knockout of KLF10 ameliorated diabetic renal fibrosis. Masson’s Trichrome staining was conducted to determine the renal fibrosis level (upper panels; left: magnification of 100×, right: magnification of 1000×). Lower panel: data are presented as means ± standard deviation from three different mice **: *p* < 0.01 compared with the control group. ##: *p* < 0.01 compared with the DM group. Scale bar: 200 μM at 100× and 20 μM at 1000×.

Furthermore, to test whether KLF10 involved in diabetes-induced renal fibrosis, we have performed Masson’s Trichrome staining and positive intensity was determined as previously reported [23]. As shown in Figure 5, Masson’s Trichrome staining intensity significantly increased in DM group compared to control group. The Masson’s Trichrome staining intensity was reduced to near control level in KLF10 knockout groups with or without DM.

**Figure 6:**
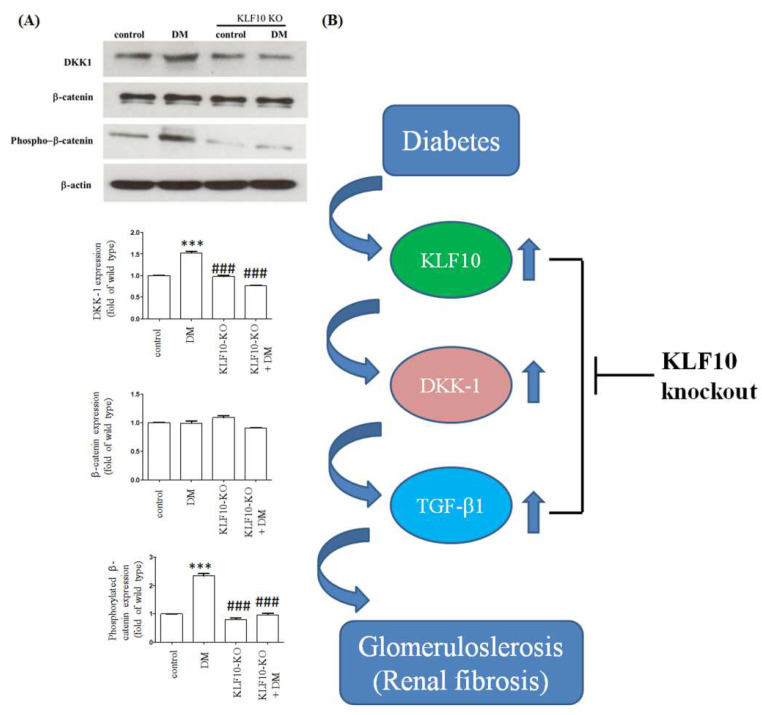
Knockout of KLF10 mitigated diabetes-induced DKK-1 and phosphorylated β-catenin expression. (**A**) Western blot analysis was conducted using proteins extracted from the kidney of control or KLF10 knockout mice with or without diabetes (DM). Data are presented as means ± standard deviation obtained from three different mice. ***: *p* < 0.001 compared with the control group. ###: *p* < 0.001 compared with the DM group. (**B**) KLF10 participated in renal fibrogenesis through the regulation of DKK-1 and its downstream pathway.

We performed Western blot analysis to further determine the effects of KLF10 knockout on fibrosis protein expression. The expression levels of DKK-1 and phosphorylated β-catenin were obviously elevated in the diabetes group compared with the normal control group. The expression of diabetes-induced DKK-1 and phosphorylated β-catenin was mitigated in the KLF10 knockout group. The expression of total β-catenin was not changed (Figure 6).

## 3. Discussion

Diabetes-induced renal fibrosis (DN) is one of the leading causes of end-stage renal disease (ESRD) in the world [15]. TGF-β1 plays a critical role in DN via the regulation of fibrosis-related protein expression. The current study demonstrated that the depletion of KLF10 repressed the expression of fibrosis-related proteins, such as TGF-β1, fibronectin, type IV collagen, phosphorylated β-catenin, and DKK-1. 

As a TGF-β1 downstream target, KLF10 participates in a wide range of cellular functions, such as cell proliferation, apoptosis, and migration [24]. Reports have shown that KLF10 is involved in fibrogenesis in several tissues. Depletion of KLF10 induces oxidative stress, enhances inflammation, and consequently causes apoptosis in high-sucrose-treated rat liver tissues [25]. Moreover, the depletion of KLF10 activates the TGF-β-Smad3 pathway and induces fibrosis, as evidenced by the accumulation of α-smooth muscle actin and collagen in high-sucrose-treated rat liver tissues [25]. Overexpression of KLF10 has been found in keloid tissues [9]. Knockout of KLF10 represses type I and III collagen, fibronectin, and matrix metalloproteinase 9 and inhibits the migration of keloid fibroblast cells [9]. In addition, Wahab et al. [26] demonstrated that connective tissue growth factor-induced KLF10 expression triggers the sustained activation of TGF-β1 involved in the progression of renal fibrosis [26]. In the present study, we demonstrated that knockout of KLF10 mitigated the expression of TGF-β1, fibronectin, and type IV collagen in diabetic glomerular mesangium. Our findings indicate that KLF10 plays a critical role in diabetic renal dysfunction through the regulation of the TGF-β1 pathway.

The role of DKK-1 in renal fibrosis remains controversial. Akhmetshina et al. [27] found the overexpression of Wnt and downregulation of DKK-1 in samples from human fibrotic diseases. In addition, forced expression of DKK-1 significantly mitigated TGF-β1-induced fibrosis in the animal model [27]. Another study used a hydrodynamic-based gene delivery method to amplify the DKK1 expression, which significantly diminished the level of β-catenin and its target genes in obstructed kidneys [18]. Furthermore, DKK-1 represses fibroblast-specific protein 1, type I collagen, and fibronectin and subsequently attenuates renal fibrosis in obstructed kidney [18]. Meanwhile, Lin et al. [21] observed elevated DKK-1 in renal mesangial cells treated with high glucose (35 mM). The overexpression of DKK-1 increased the TGF-β1 and fibronectin level, whereas knockout of DKK-1 repressed these proteins’ expression [21]. DKK-1 also attenuated nuclear localization and increased the phosphorylation of β-catenin [21]. In line with this observation, we obtained a significantly high expression of DKK-1 in the STZ-treated diabetic mice, whereas knockout of KLF10 reduced DKK-1 expression. Our results indicate that DKK-1 may mediate KFL10-induced fibrogenesis in diabetes conditions.

## 4. Materials and Methods

### 4.1. Animal Model

KLF10 knockout mice were generated in accordance with previous reports [28]. In this study, 4-month-old C57BL/6 mice were divided into four subgroups: (1) control, (2) KLF10 knockout (KLF-10-KO), (3) control plus STZ treatment (DM), and (4) KLF-10-KO treatment with STZ (KLF-10-KO-DM). Each subgroup contained six mice. To induce diabetes, the mice were intraperitoneally administered 50 mg/kg of STZ. A fasting blood glucose level above 200–300 mg/dL was regarded as a symptom of diabetes. To equalize the blood glucose, the diabetic mice were subcutaneously administered with 1–2 unit/kg of insulin once a day. All the animals were sacrificed after five weeks. The urine protein and creatinine and serum glucose and HbA1C level were measured as previously reported [29]. The animal procedures were approved by the Institutional Animal Care and Use Committee of Chang Gung Memorial Hospital.

### 4.2. Immunohistochemical (IHC) and Masson’s Trichrome Staining

The 4 μm paraffin-embedded renal tissue sections were deparaffinized and rehydrated. The sections were treated with 3% H_2_O_2_ to remove endogenous peroxidase activity. Antigens were retrieved by boiling for 20 min in 10 mM of citrate buffer (pH 6.0). After incubation with the antibody, the immunohistochemical signals were detected using a horseradish peroxidase-3-3-diaminobenzidene kit (BioGenex, San Ramon, CA, USA), followed by hematoxylin counterstaining. Masson’s Trichrome staining was conducted as previously reported [23]. IHC and Masson’s Trichrome staining intensity were captured in six randomly selected regions of three sections from three mice and measured using Image-Pro^®^ Plus image analysis software (Media Cybernetics, Silver Spring, MD, USA). The fibronectin (F2372) and TGFβ1 (Y369) antibodies were obtained from Bioworld Technology (Nanjing, China). DKK1 (sc-25516) was obtained from Santa Cruz Biotechnology (Santa Cruz, CA, USA). Phospho-β-Catenin (Ser675) (D2F1) (#4176) was purchased from Cell Signaling Technology (Danvers, MA, USA). Anti-Collagen IV antibody (ab6586) was purchased from Abcam (Cambridge, UK).

### 4.3. Laser Capture Microdissection

Renal tissues from different groups were dissected and longitudinally cut into 4 μm thick sections. Two hundred glomerular mesangium from six sections were captured with the VERITAS™ laser-captured dissection system (Arcturus Bioscience Inc., Mountain View, CA, USA) in accordance with the manufacturer’s protocols.

### 4.4. Quantitative Reverse Transcription–Polymerase Chain Reaction

Total RNA was extracted from glomerular mesangium by using the TRI reagent. Then, 1 μg of total RNA was reversed transcribed into the first strand cDNA by using mirVana 5 × RT Buffer, 1 × mirVana specific RT primers, and ArrayScript Enzyme Mix (Ambion Inc., Austin, TX, USA). Quantitative polymerase chain reaction (PCR) analysis was conducted in 25 μL mixtures containing the cDNA template, primers, and iQ SYBR Green Supermix amplified in an iCycler iQ real-time PCR detection system (Bio-RadLaboratories, Hercules, CA, USA), as previously described [23]. The primer sequences were as follows: TGF-β1 (forward 5′-TGA GTG GCT GTC TTT TGA CG-3′; reverse 5′-TGG GAC TGA TCC CAT TGA TT-3′), fibronectin (forward 5′-GTG GCT GCC TTC AAC TTC TC-3′; reverse 5′-AGT CCT TTA GGG CGG TCA AT-3′), DKK1 (forward 5′-TCC GTCTGC CTC CGA TCA TC-3′; reverse 5′-GCC TTT CCG TTT GTG CTT GG-3′), α1 (IV) collagen(forward, 5′-ATTCCTTTGTGATGCACACCAG-3′; Type IV collagen reverse, 5′-AAGCTGTAAGCATTCGCGTAGTA-3′), and β-actin (forward 5′-CGC CAA CCG CGA GAA GAT-3′; reverse 5′-CGT CAC CGG AGT CCA TCA-3′). Ribosome 5S was used as the internal control. The fold change was measured as 2−ΔΔCt, where ΔΔCt = ΔCttreatment − ΔCtvehicle and ΔCt = Cttarget − Ct5S.

### 4.5. Western Blot Analysis

Proteins extracted from glomerular mesangium using tissue protein extraction reagent (Pierce, Rockford, IL, USA) as previously reported [30] were separated by SDS-PAGE and electro-transferred to nitride cellulose membranes. The membranes were blocked with phosphate-buffered saline (PBS) containing 5% non-fat milk for 1 h, washed three times with PBST (PBS plus 0.1% Tween-20), and immersed in the indicated primary antibodies overnight at 4 °C. After washing with PBST three times, the membranes were incubated with horseradish peroxidase (HRP)-conjugated second antibodies at room temperature for 1 h. The positive signals were detected via enhanced chemiluminescence (ECL). Antibody against β-catenin (#9562) and β-actin (SC-4970) was purchased from Cell Signaling. βactin was used as the loading control.

### 4.6. Statistical Analysis

Data are presented as means and standard deviation. One-way ANOVA with Tukey’s post hoc test was applied to detect the difference between groups by using GraphPad Prism 5 software. A *p* value < 0.05 was set to be significant.

## 5. Conclusions

Collectively, our results show that the interference of KLF10 expression repressed DKK-1 expression and abolished the expression of TGF-β1 and fibrosis-related proteins, such as fibronectin and type IV collagen (Figure 6B). Decreased DKK-1 expression reduced the phosphorylated β-catenin expression (Figure 6B) [31]. In conclusion, KLF10 mediated DKK-1 expression played a pivotal role in high-glucose-induced renal fibrosis. Our findings suggest that the target of KLF10 may be a potential therapeutic method to prevent diabetic renal fibrosis. However, it is not feasible to translate this to a human model, because the knockout of KLF10 may affect several human diseases such as cancer, cardiovascular disease, and osteoporosis. In the future, the development of specific small interference RNA for knockout of KLF10 may be a promising clinical therapy for renal fibrosis.

## Figures and Tables

**Table 1:** The biochemical properties of experimental animals (n = 6).

	Control	DM	KLF10-KO	KLF10-KO + DM
Fasting blood glucose (mg/dL)	156 ± 21	487 ± 70 *	153 ± 17	496 ± 56 *
HbA1C (%)	4.1 ± 0.9	8.1 ± 0.9 *	4.3 ± 1.2	8.4 ± 0.3 *
Body weight (BW, g)	29.1 ± 1.4	24.1 ± 0.7 *	33.0 ± 1.9	23.9 ± 1.2 *
Kidney/BW (%)	0.76 ± 0.21	1.10 ± 0.21 *	0.82 ± 0.23	0.89 ± 0.07 *#
Urine (TP/CRE)	5.9 ± 0.7	11.2 ± 1.2	6.1 ± 0.4	8.4 ± 0.7 *#

TP: total protein, CRE: creatinine. *: *p* < 0.05 compared to control; #: *p* < 0.05 compared to DM group. BW: body weight.
